# Supplementary material for: Acylated heptapeptide binds albumin with high affinity and application as tag furnishes long-acting peptides
Source: Nat Commun. 2017 Jul 17;8:16092. doi: 10.1038/ncomms16092 (PMC5520048; doi:10.1038/ncomms16092)
Supplement: Supplementary Information [file ncomms16092-s1.pdf]

SI GUIDE

Type of file: pdf
Size of file: 3,353 KB
Title of file for HTML: Supplementary Information
Description: Supplementary Figures and Supplementary Tables.

Type of file: pdf
Size of file: 477 KB
Title of file for HTML: Peer Review File
Description:.

Supplementary Tables

	albumin binding (K_d) [nM]		FXIIa inhibition (K_i) [nM]	
	human serum	rabbit albumin	no albumin	with albumin
tag-FXIIa inhibitor	173 ± 19	650 ± 80	14.9 ± 0.6	116 ± 16
tag-PEG2-FXIIa inhibitor	145 ± 9	740 ± 100	10.6 ± 1.2	68 ± 8
tag-2xPEG2-FXIIa inhibitor	185 ± 6	890 ± 120	7.3 ± 1.3	32 ± 8
tag-3x PEG2-FXIIa inhibitor	205 ± 8	1080 ± 150	9.3 ± 1.4	30 ± 4
tag-4x PEG2-FXIIa inhibitor	218 ± 3	1220 ± 190	5.6 ± 1.4	16 ± 3
tag-5x PEG2-FXIIa inhibitor	219 ± 7	1400 ± 200	6.6 ± 1.8	13 ± 1
tag-3xPEG24-FXIIa inhibitor	224 ± 11	1600 ± 300	3.1 ± 0.6	4 ± 0.9
FXIIa inhibitor-tag	138 ± 14	510 ± 60	2.2 ± 0.5	7 ± 3
FXIIa inhibitor-PEG2-tag	142 ± 8	680 ± 90	2.1 ± 0.5	5.8 ± 1.9
FXIIa inhibitor-3xPEG2-tag	177 ± 7	880 ± 130	2.5 ± 0.2	5.8 ± 1.7

Supplementary Table 1. Albumin binding and inhibition activity of tagged bicyclic peptide FXIIa inhibitor. The tag was conjugated via different linkers to either the N- or C-terminus of the bicyclic peptide FXIIa inhibitor. PEG2 and PEG24 stand for linkers containing 2 or 24 CH_2CH_2O units. Non-tagged FXIIa inhibitor was labeled with fluorescein at the N-terminus. Binding constants K_ds for human albumin in serum and rabbit albumin were measured by fluorescence polarization. Inhibition constants K_is for FXIIa were measured in an enzymatic assay using fluorogenic FXIIa substrate in presence and absence of human albumin (10 μM). Standard deviations of at least three measurements are indicated.

	$EC_{1.5x}$ aPTT human plasma (μM)	EC_{5x} aPTT rabbit plasma (μM)
FXIIa inhibitor	0.18 ± 0.06	0.11 ± 0.03
tag-5xPEG2-FXIIa inhibitor	4.4 ± 0.3	> 10
tag-3xPEG24-FXIIa inhibitor	5.1 ± 0.4	4.2 ± 0.5
FXIIa inhibitor-3xPEG2-tag	6.8 ± 1.8	> 10

Supplementary Table 2. Inhibition of the intrinsic coagulation pathway by the bicyclic peptide FXIIa inhibitor modified at either end with the tag. The aPTT was measured in human and rabbit plasma at different concentrations of the inhibitor conjugates and the concentrations calculated at which the aPTT is increased 50% (human plasma; $EC_{1.5x}$) or 500% (rabbit plasma; EC_{5x}). Note: Inhibition of FXIIa affects the intrinsic coagulation time to a larger extent in rabbit plasma than in human plasma. For rabbit plasma, an EC_{5x} is thus indicated instead of an $EC_{1.5x}$.

	tag-3xPEG24-FXIIa inhibitor	FXIIa inhibitor
$t_{1/2}$ α (hrs)	1.0 ± 0.2	n.d.
$t_{1/2}$ β (hrs)	5.2 ± 0.4	0.21 ± 0.04
AUC (mg min ml^{-1})	41 ± 7	0.18 ± 0.01
CL (ml hr^{-1} kg)	6 ± 1	1000 ± 40
V_D (ml kg^{-1})	46 ± 7	300 ± 50

Supplementary Table 3. Pharmacokinetic parameters of tagged and non-tagged FXIIa inhibitor in rabbits. Average values and standard deviations are indicated (n = 3).

Supplementary Figures

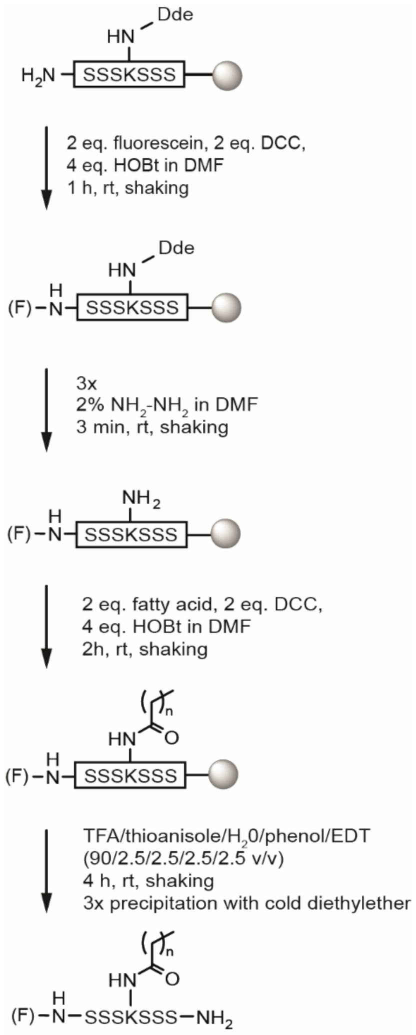

Supplementary Figure 1. Solid phase synthesis strategy for peptides acylated with a fatty acid. A peptide containing six serine residues and a central lysine is synthesized using standard Fmoc chemistry. The side chain of lysine is protected with Dde. Fluorescein is appended via a PEG linker [(CH_2CH_2O)$_2$] to the last amino acid at the N-terminus of the peptide through formation of a peptide bond. Subsequently, the Dde protection group of lysine is selectively removed by reaction with hydrazine and a fatty acid is conjugated to the ε-amino group by formation of a peptide bond using DCC as coupling reagent. The peptide is cleaved from the resin and the side chains are deprotected using a standard cleavage cocktail.

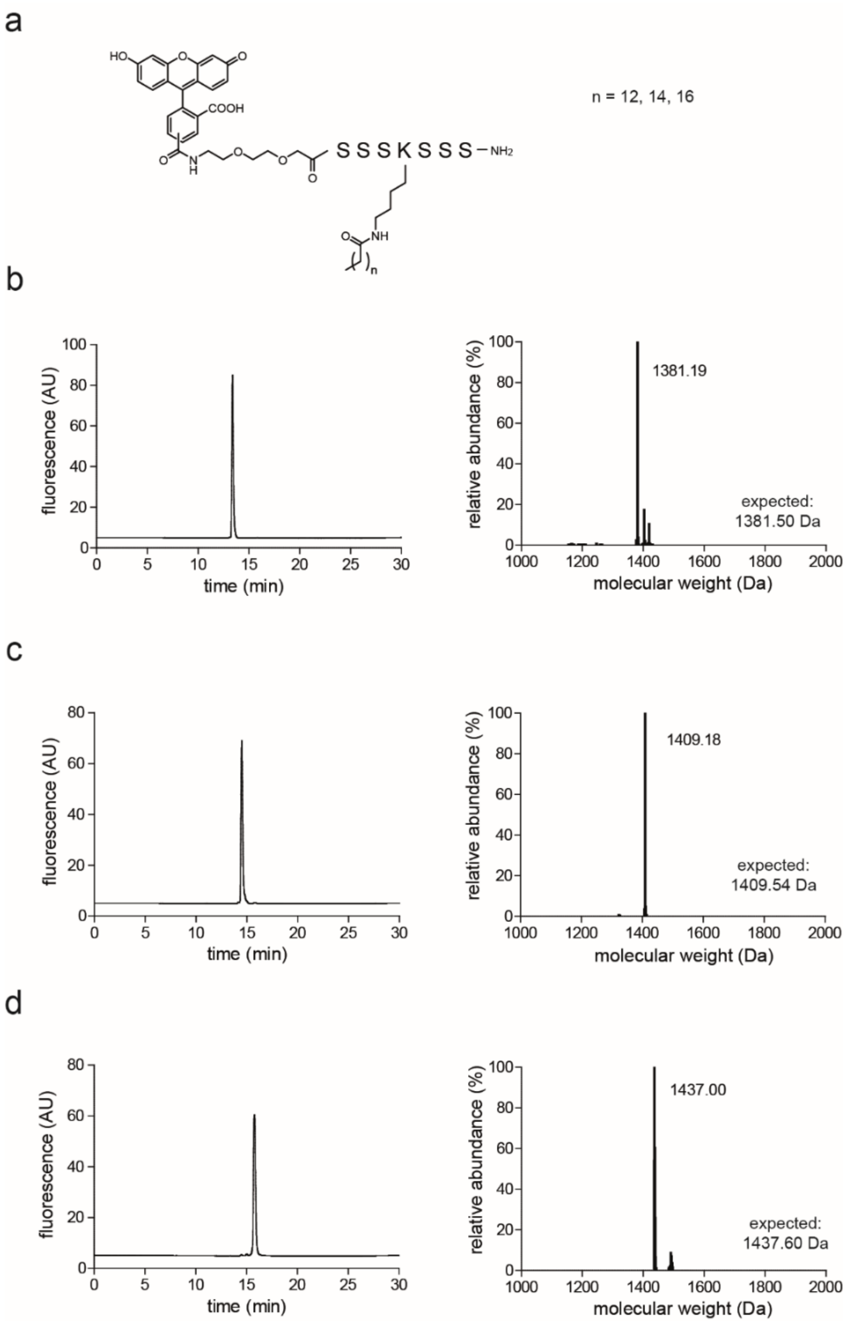

Supplementary Figure 2. Peptide SSSKSSS carrying a fluorescein at the N-terminus and a fatty acid at the side chain of the lysine. The chemical structure is shown in **(a)** and the RP-HPLC chromatograms as well as mass spectra are shown below for the peptide carrying myristic acid **(b)**, palmitic acid **(c)** and stearic acid **(d)**.

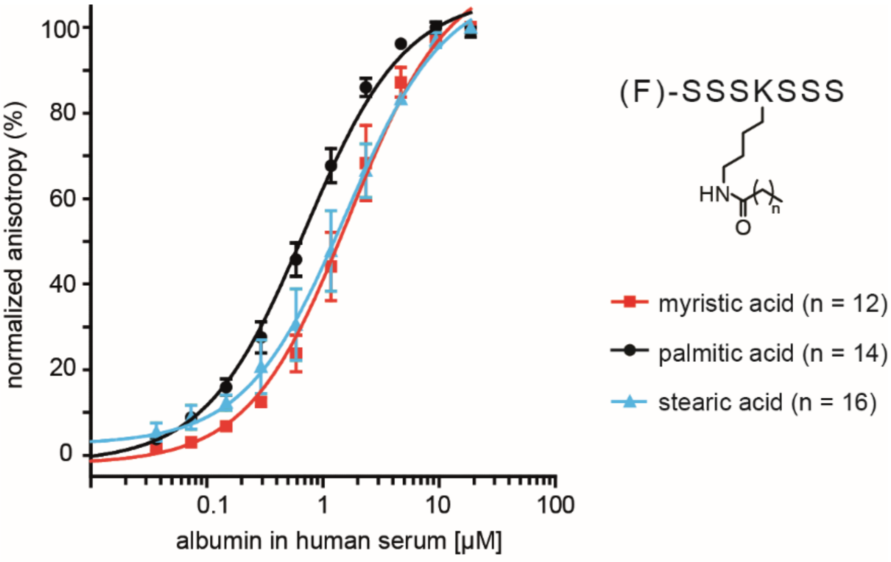

	albumin binding (K_d) [nM]	
	human albumin	human serum
(F)-SSSK$_{myr}$SSS	1900 ± 200	1680 ± 190
(F)-SSSK$_{palm}$SSS	1080 ± 120	690 ± 70
(F)-SSSK$_{ste}$SSS	1480 ± 150	1560 ± 100

Supplementary Figure 3. Binding of the fluorescein labeled peptide SSSKSSS lipidated with myristic, palmitic or stearic acid to human albumin. The dissociation constants were determined by measuring fluorescence polarization of peptide incubated with human serum that is diluted to different extent with PBS and thus contains human albumin at different concentrations. The table shows the calculated K_ds from this experiment as well as from the same experiment performed with purified human albumin (raw data shown in Figure 1c). Average values and standard deviations of three measurements are shown.

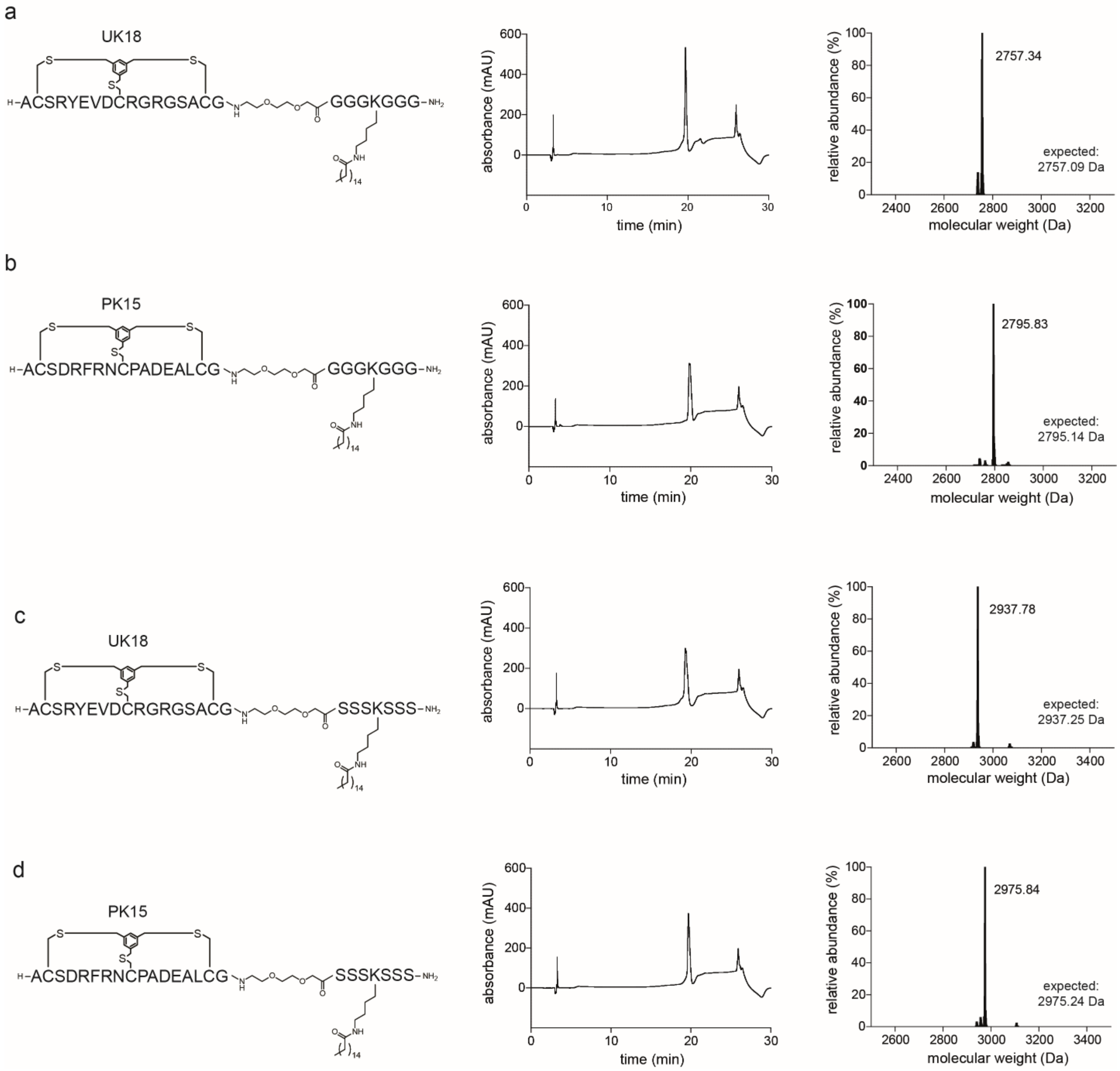

Supplementary Figure 4. Chemical structures, RP-HPLC chromatograms and mass spectra of bicyclic peptides carrying a peptide-fatty acid tag. Bicyclic peptides UK18 **(a, c)** and PK15 **(b, d)** conjugated via their C-terminal end to the peptides GGGKGGG **(a, b)** or SSSKSSS **(c, d)** that are lipidated at the side chain of lysine with palmitic acid.

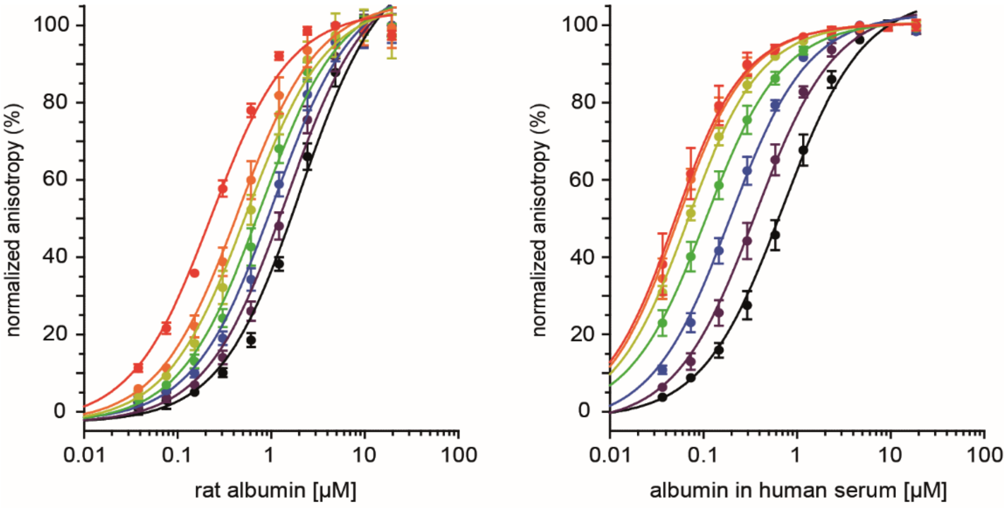

		albumin binding (K_d) [nM]		
		human albumin	rat albumin	human serum
cycle 0	(F)–SSSK$_{palm}$SSS	1080 ± 120	2000 ± 300	690 ± 70
cycle 1	(F)–SSSK$_{palm}$ESS	520 ± 50	1500 ± 200	350 ± 30
cycle 2	(F)–SSEK$_{palm}$ESS	250 ± 30	1050 ± 150	180 ± 16
cycle 3	(F)–SYEK$_{palm}$ESS	123 ± 9	770 ± 110	95 ± 3
cycle 4b	(F)–SYEK$_{palm}$ESE	76 ± 7	540 ± 70	57 ± 2
cycle 5a, 5c	(F)–EYEK$_{palm}$ESE	46 ± 4	430 ± 50	41 ± 3
cycle 6	(F)–EYEK$_{palm}$EYE	39 ± 3	220 ± 30	37 ± 2

Supplementary Figure 5. Binding of fluorescein-labeled and palmitoylated peptide identified in different evolution cycles. Binding to rat albumin and human albumin in serum samples diluted with PBS was measured by fluorescence polarization. K_ds are indicated in the table along with standard deviations calculated based on three measurements. K_ds for the same experiment performed with purified human albumin (raw data shown in Figure 2b) are included. Average values and standard deviations of three measurements are shown.

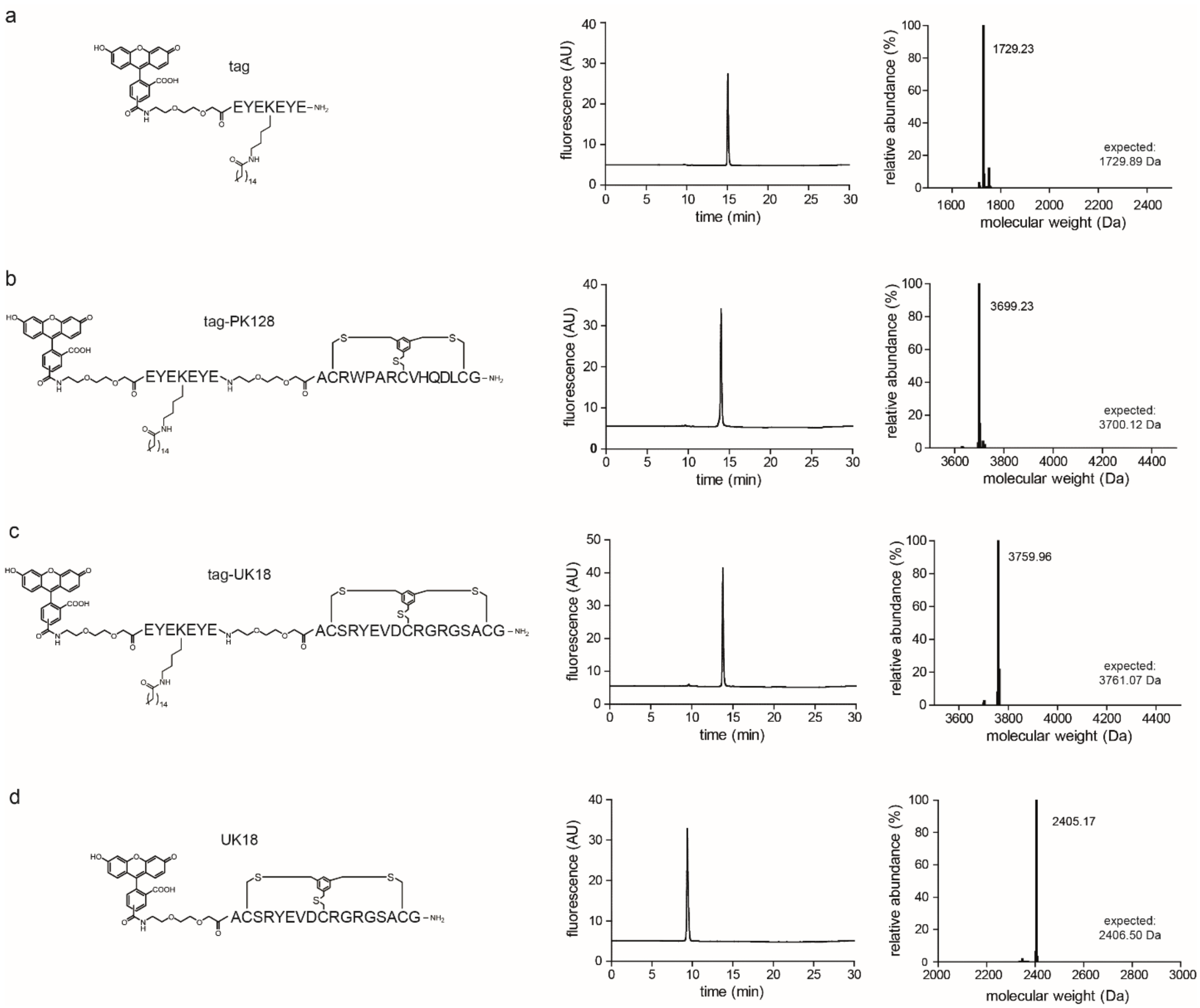

Supplementary Figure 6. Chemical structures, RP-HPLC chromatograms and mass spectra of the tag, tagged bicyclic peptides and fluorescein-labeled bicyclic peptide. Tag **(a)**, tagged bicyclic peptide PK128 (tag-PK128) **(b)**, tagged bicyclic peptide UK18 (tag-UK18) **(c)** and fluorescein-labeled bicyclic peptide UK18 **(d)**.

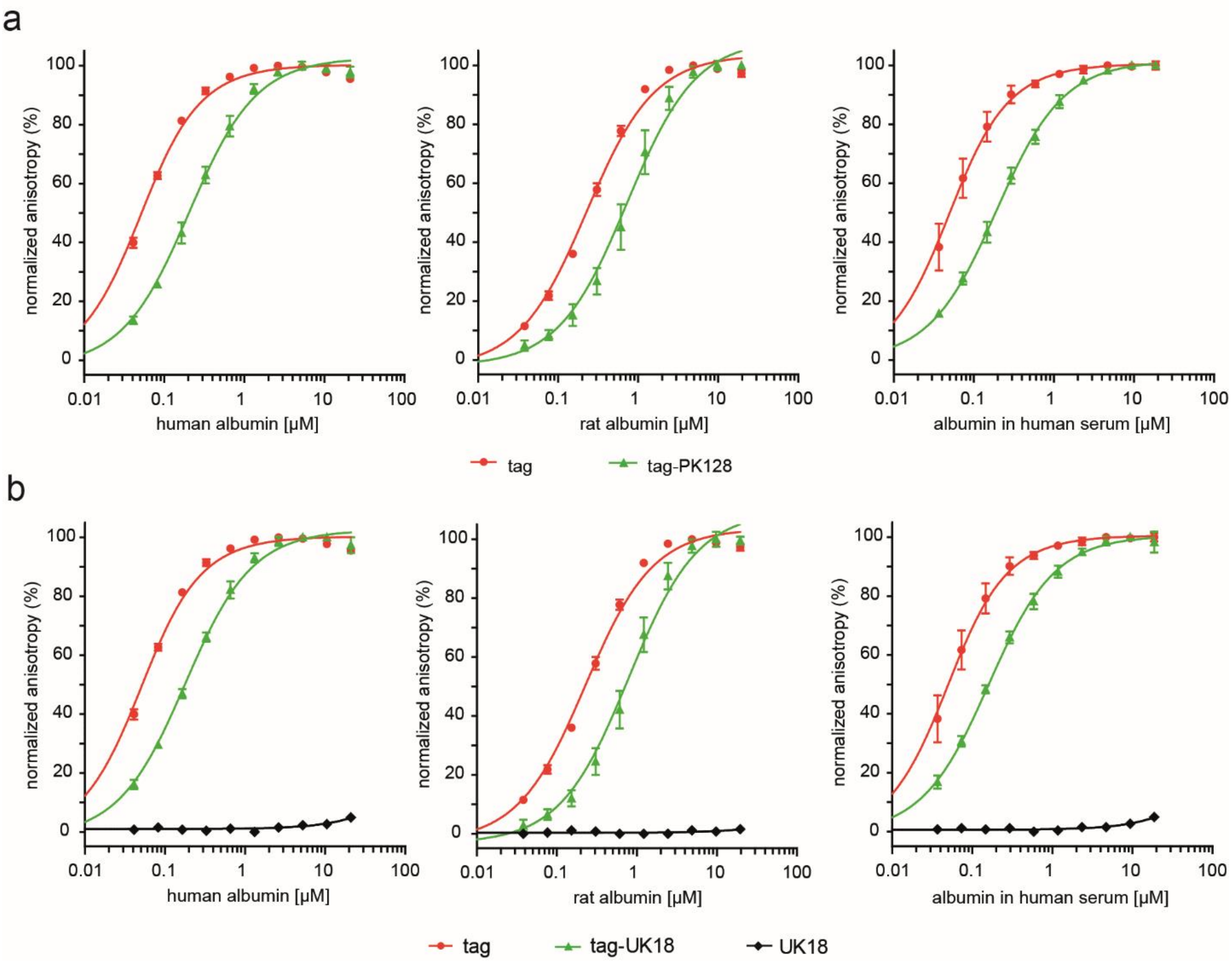

Supplementary Figure 7. Binding of tagged bicyclic peptides to human albumin, rat albumin and human albumin in serum. Human serum was diluted with PBS. Binding was measured by fluorescence polarization. **(a)** Comparison of tag and tag-PK128. **(b)** Comparison of tag, tag-UK18 and UK18. Average values and standard deviations of three measurements are shown.

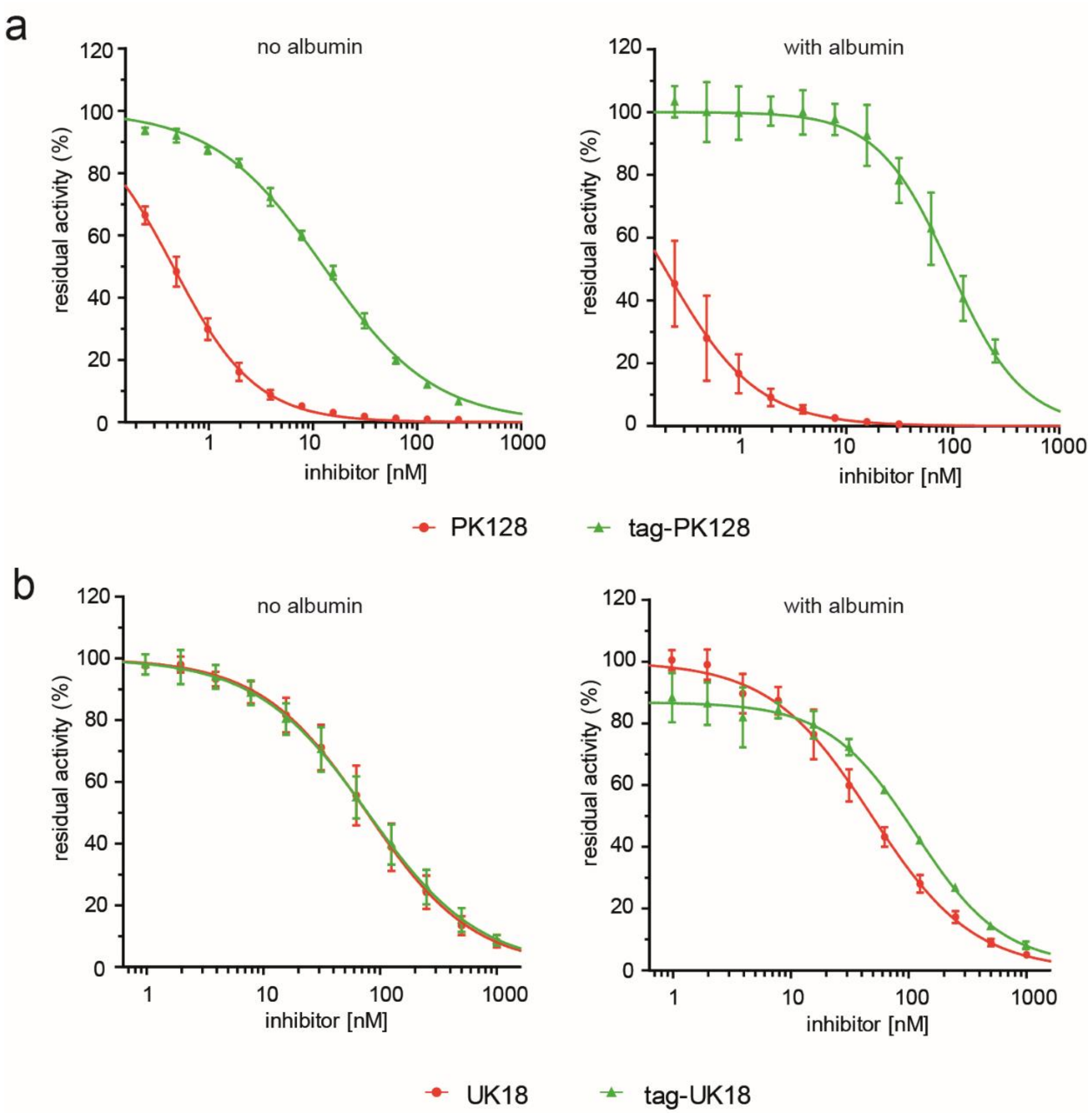

Supplementary Figure 8. Inhibitory activity of tag-bicyclic peptide conjugates in presence and absence of human albumin (25 μM). (a) Comparison of PK128 and tag-PK128. Residual activity of human plasma kallikrein was measured with the fluorogenic substrate Z-Phe-Arg-AMC. **(b)** Comparison of UK18 and tag-UK18. Residual activity of human urokinase was measured with the fluorogenic substrate Z-Gly-Gly-Arg-AMC. Average values and standard deviations of three measurements are shown.

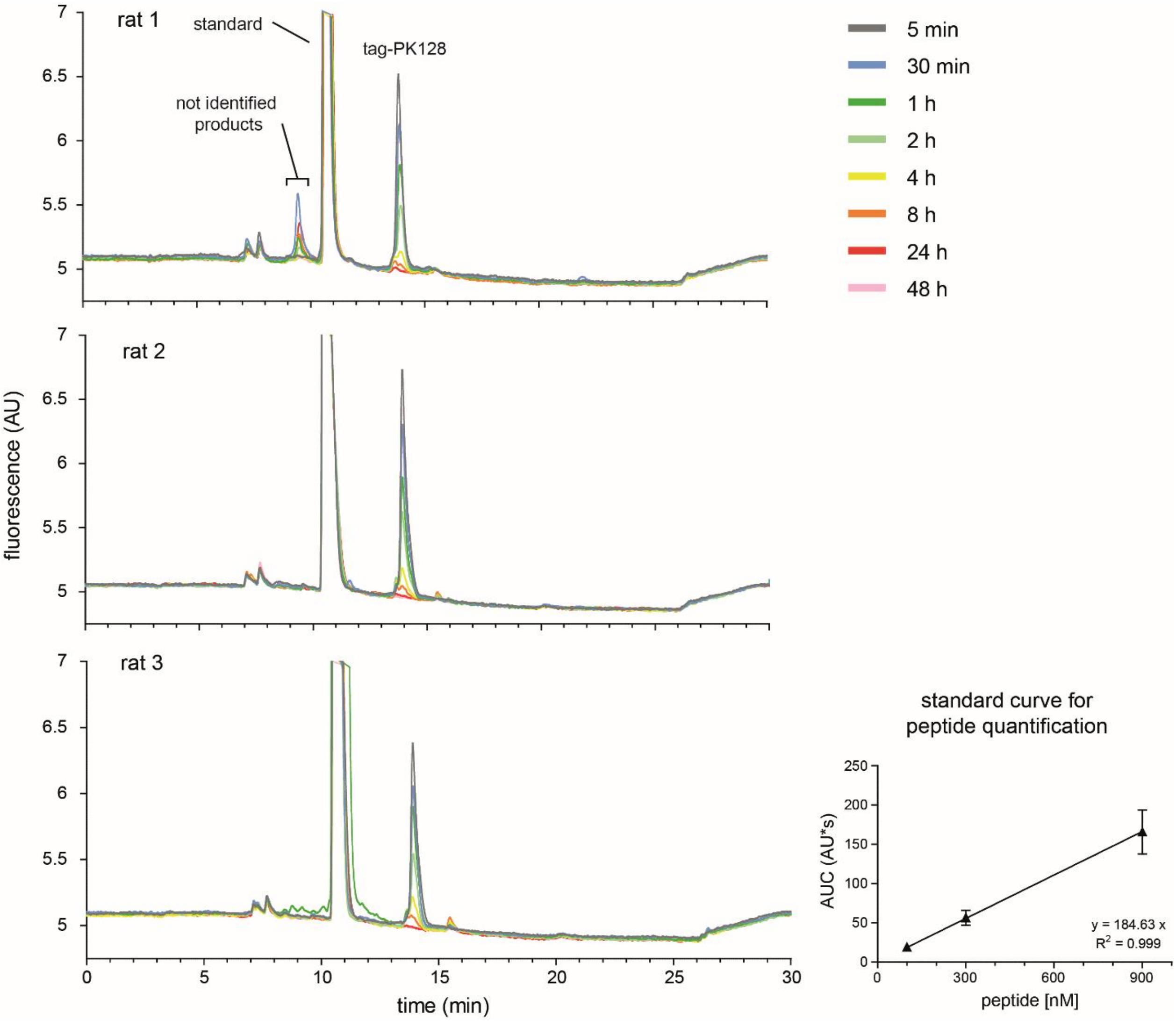

Supplementary Figure 9. Pharmacokinetic study of tag-PK128 in rats. Fluorescent peptide species in blood samples taken from three rats at the indicated time points were analyzed by RP-HPLC and fluorescence detection. The blood samples were spiked with fluorescein as standard. Average values and standard deviations of three measurements for standard curve preparation are shown.

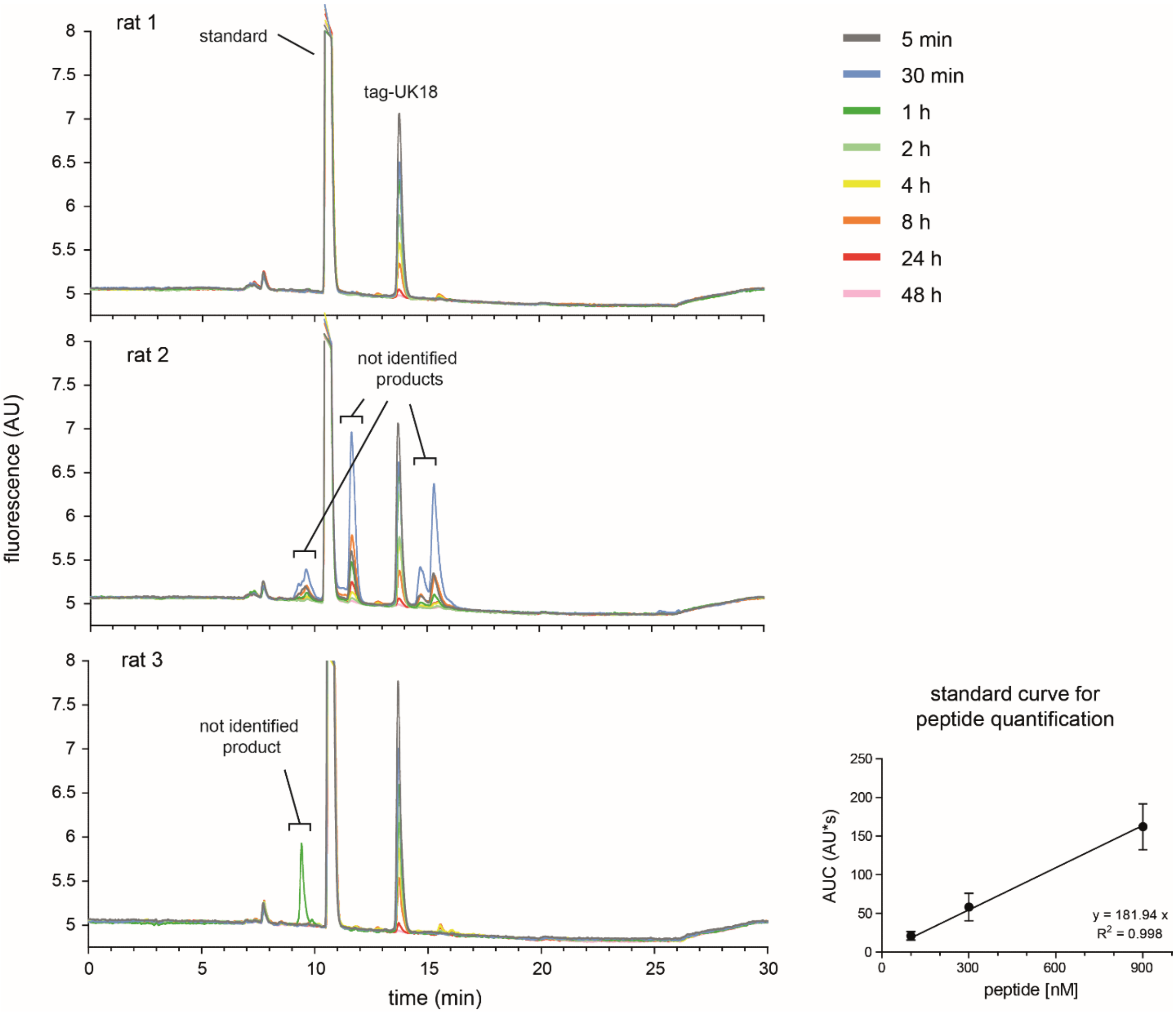

Supplementary Figure 10. Pharmacokinetic study of tag-UK18 in rats. Fluorescent peptide species in blood samples taken from three rats at the indicated time points were analyzed by RP-HPLC and fluorescence detection. The blood samples were spiked with fluorescein as standard. Average values and standard deviations of three measurements for standard curve preparation are shown.

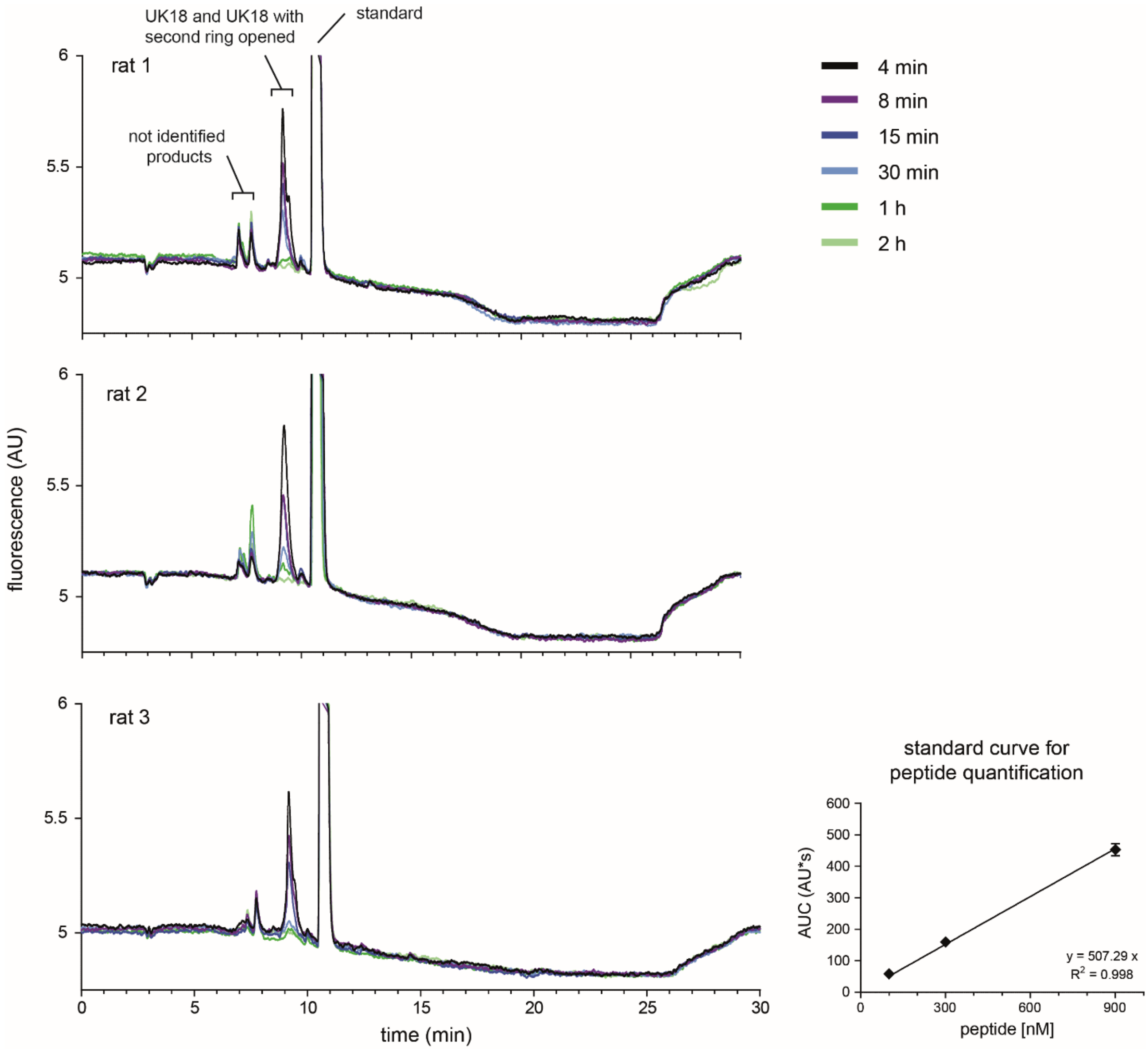

Supplementary Figure 11. Pharmacokinetic study of fluorescein labeled bicyclic peptide UK18 in rats. Fluorescent peptide species in blood samples taken from three rats at the indicated time points were analyzed by RP-HPLC and fluorescence detection. The blood samples were spiked with fluorescein as standard. Average values and standard deviations of three measurements for standard curve preparation are shown.

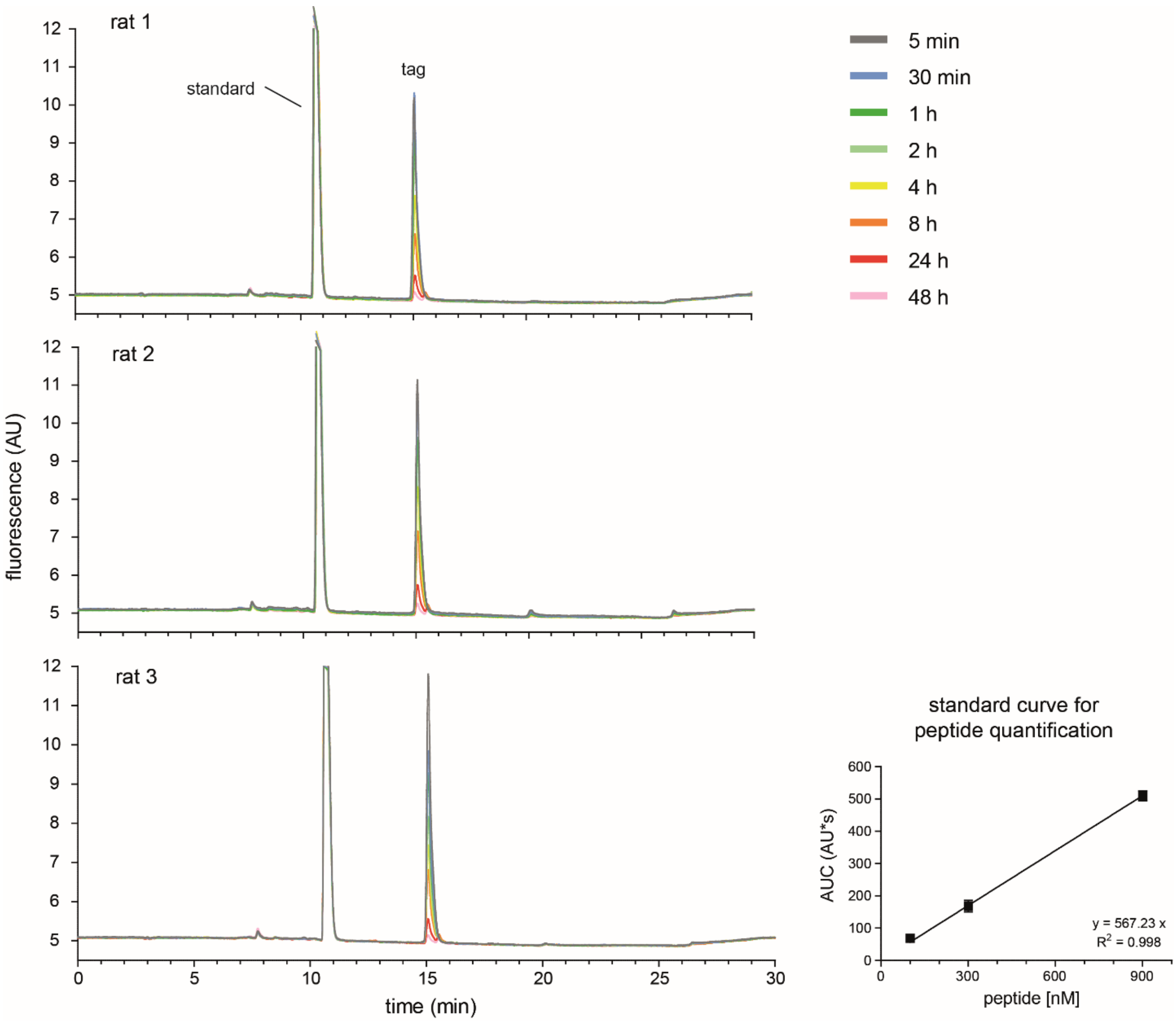

Supplementary Figure 12. Pharmacokinetic study of the tag in rats. Fluorescent peptide species in blood samples taken from three rats at the indicated time points were analyzed by RP-HPLC and fluorescence detection. The blood samples were spiked with fluorescein as standard. Average values and standard deviations of three measurements for standard curve preparation are shown.

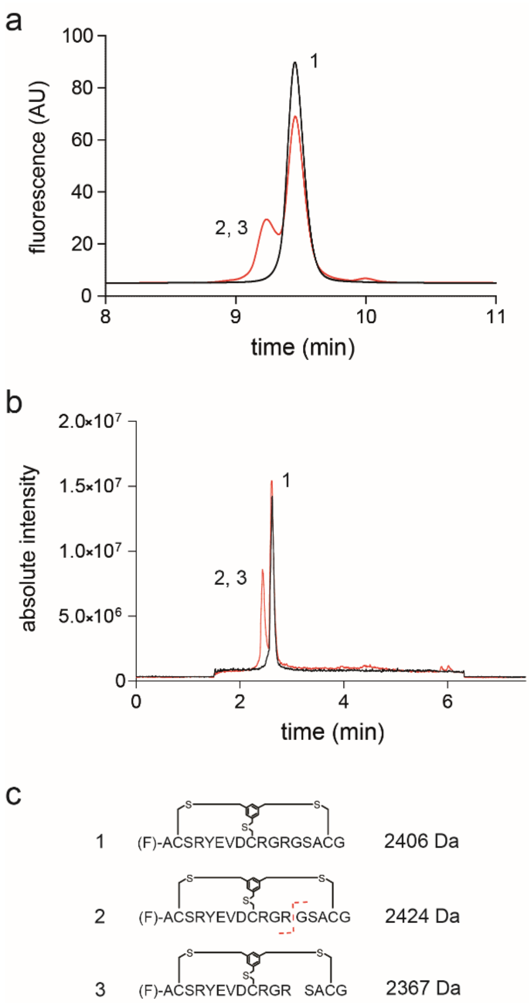

Supplementary Figure 13. Proteolytic stability of UK18 in rat plasma *ex vivo*. (a) Fluorescein labeled UK18 (1) incubated in rat plasma at 37 °C for three days was analyzed by RP-HPLC and fluorescence detection in order to identify potential degradation products. **(b)** LC-MS analysis of purified peaks. **(c)** Potential products (2, 3) that correspond to the observed masses.

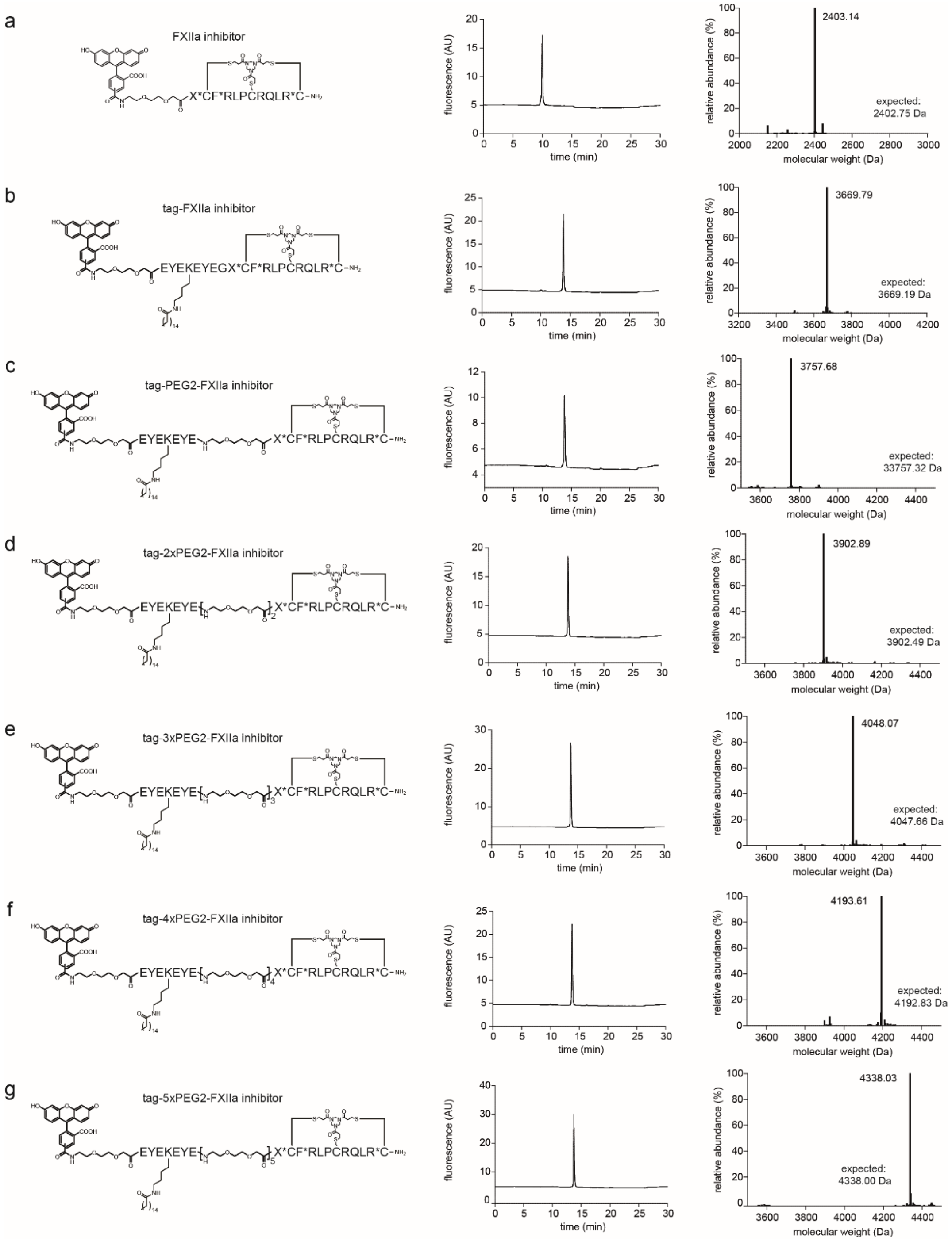

Supplementary Figure 14. Bicyclic peptide FXIIa inhibitor conjugated via different linkers to the tag. Chemical structures, analytical RP-HPLC chromatograms and mass spectra of bicyclic peptide FXIIa inhibitor conjugated via different linkers to the tag.

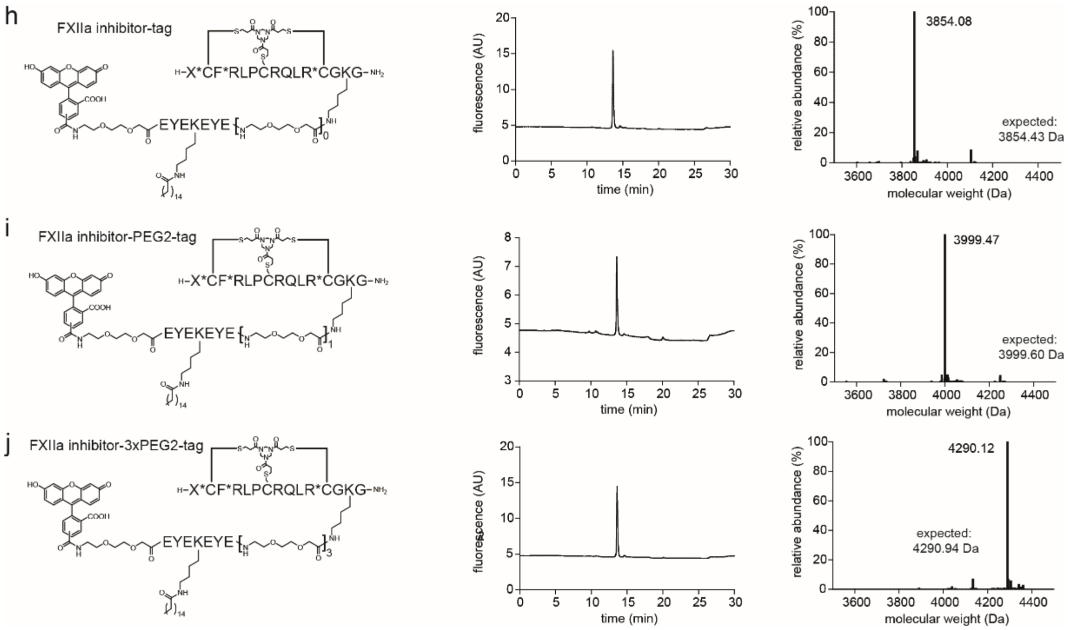

Supplementary Figure 14. (continued)

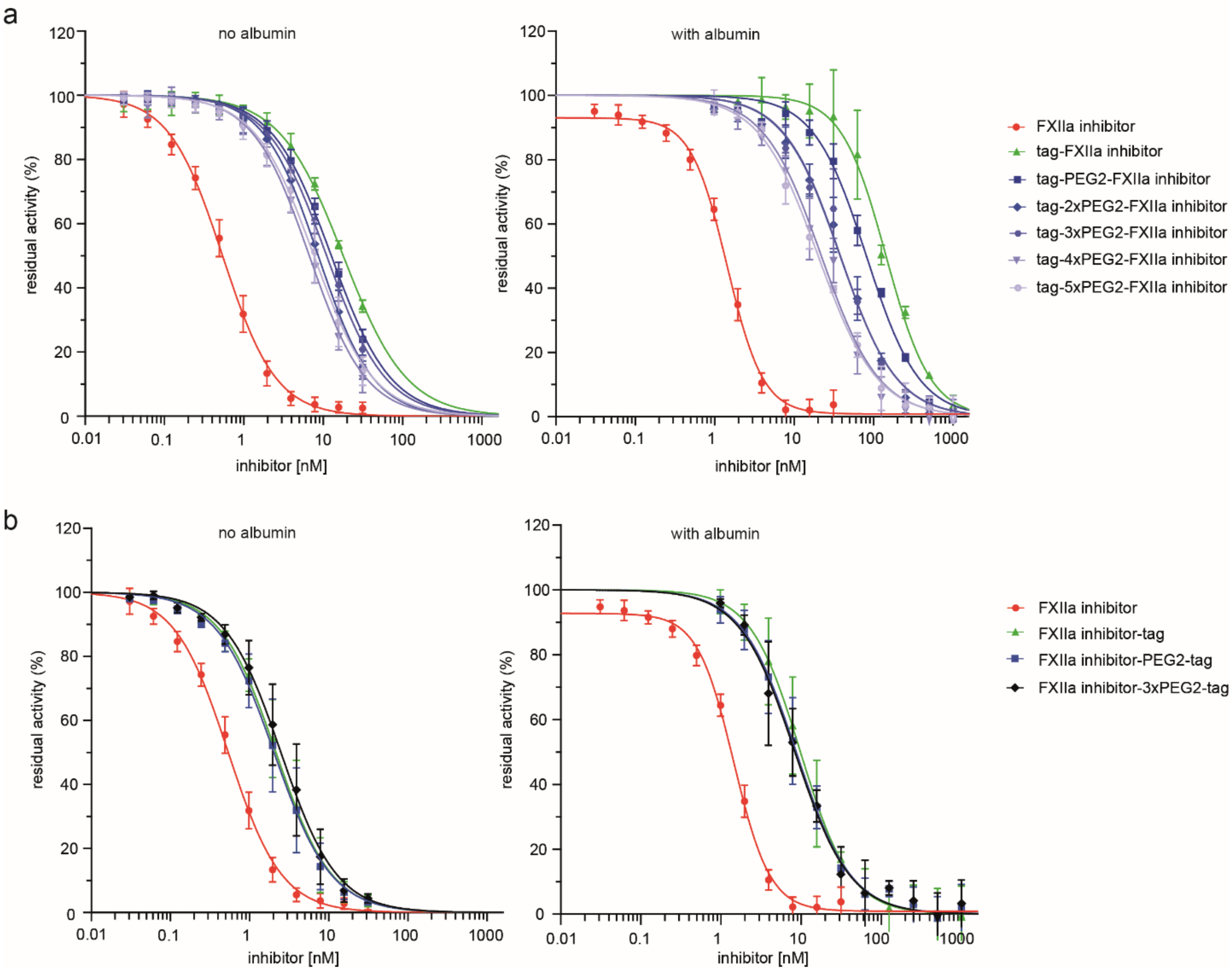

Supplementary Figure 15. Inhibitory activity of bicyclic peptide FXIIa inhibitor conjugated via different linkers to the tag. Residual activity of FXIIa was measured with the fluorogenic substrate Boc-Gln-Gly-Arg-AMC in presence and absence of human albumin (10 μM). **(a)** Conjugates with the tag linked to the N-terminal end of the bicyclic peptide. **(b)** Conjugates with the tag linked to the C-terminal end of the bicyclic peptide. Average values and standard deviations of three measurements are shown.

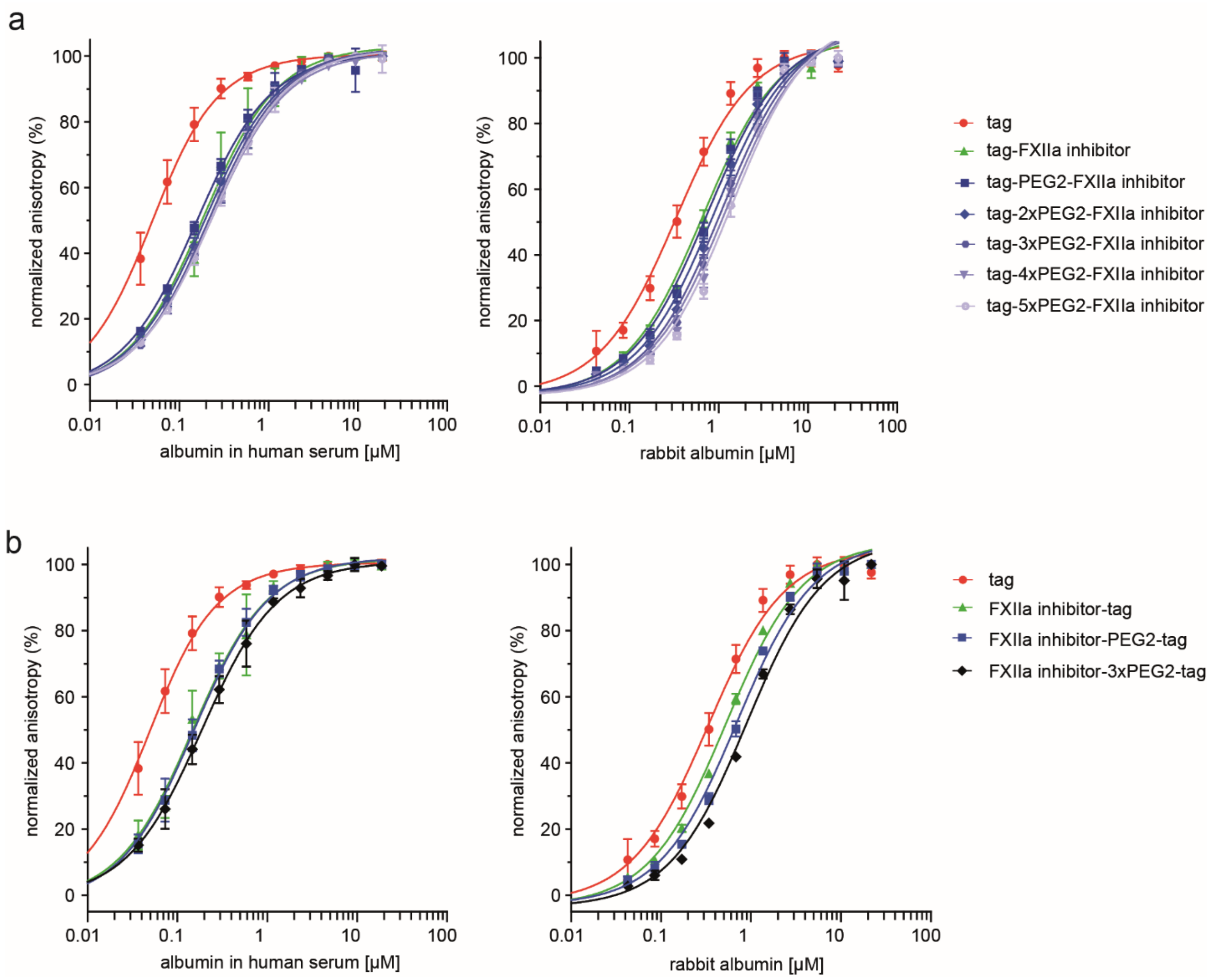

Supplementary Figure 16. Binding affinity of bicyclic peptide FXIIa inhibitor conjugated via different linkers to the tag for human albumin in serum and rabbit albumin. Human serum was diluted with PBS. Binding was measured by fluorescence polarization. **(a)** Conjugates with the tag linked to the N-terminal end of the bicyclic peptide. **(b)** Conjugates with the tag linked to the C-terminal end of the bicyclic peptide. Average values and standard deviations of three measurements are shown.

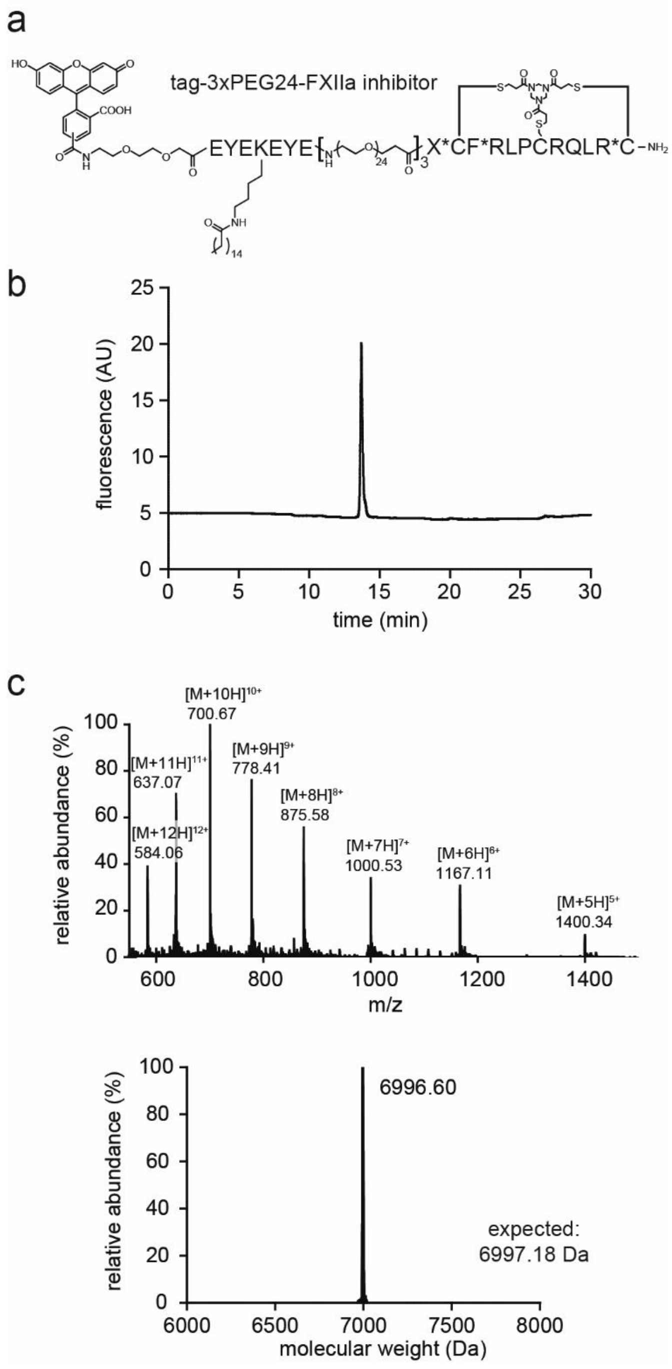

Supplementary Figure 17. Bicyclic peptide FXIIa inhibitor conjugated via a long PEG linker to the albumin tag. Chemical structure **(a)**, analytical RP-HPLC chromatogram **(b)** and mass spectrum before and after deconvolution **(c)** of bicyclic peptide FXIIa inhibitor conjugated via a long PEG linker to the tag.

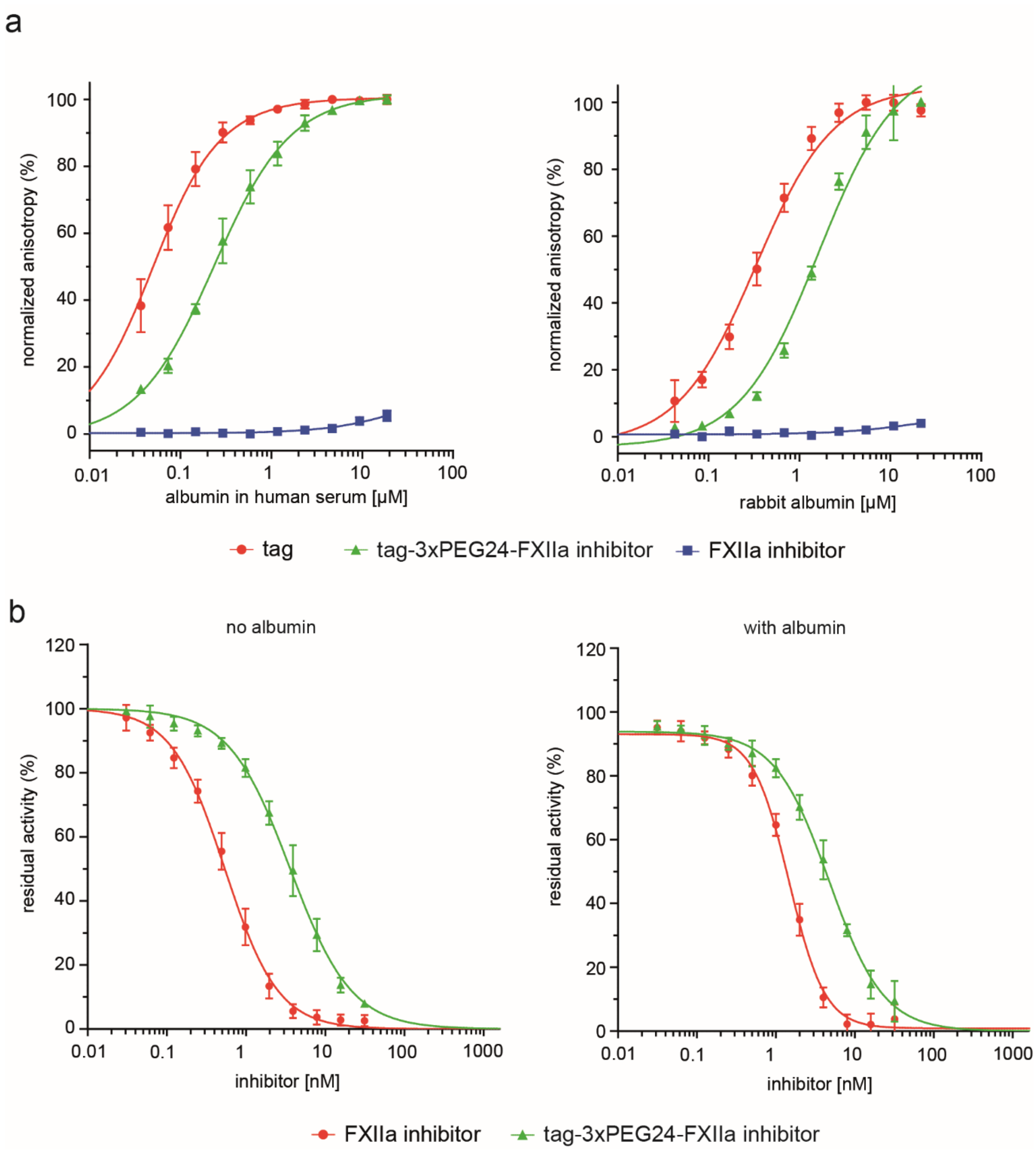

Supplementary Figure 18. Albumin binding and FXII inhibition activity of the tag-3xPEG24-FXIIa inhibitor. (a) Binding to human albumin in serum samples diluted with PBS and rabbit albumin was measured for tag alone, tag-3xPEG24-FXIIa inhibitor and FXIIa inhibitor by fluorescence polarization. **(b)** The inhibition constants of FXIIa inhibitor and tag-3xPEG24-FXIIa were determined by measuring residual FXIIa activity with the fluorogenic substrate Boc-Gln-Gly-Arg-AMC in presence and absence of human albumin (10 μM). Average values and standard deviations of three measurements are shown.

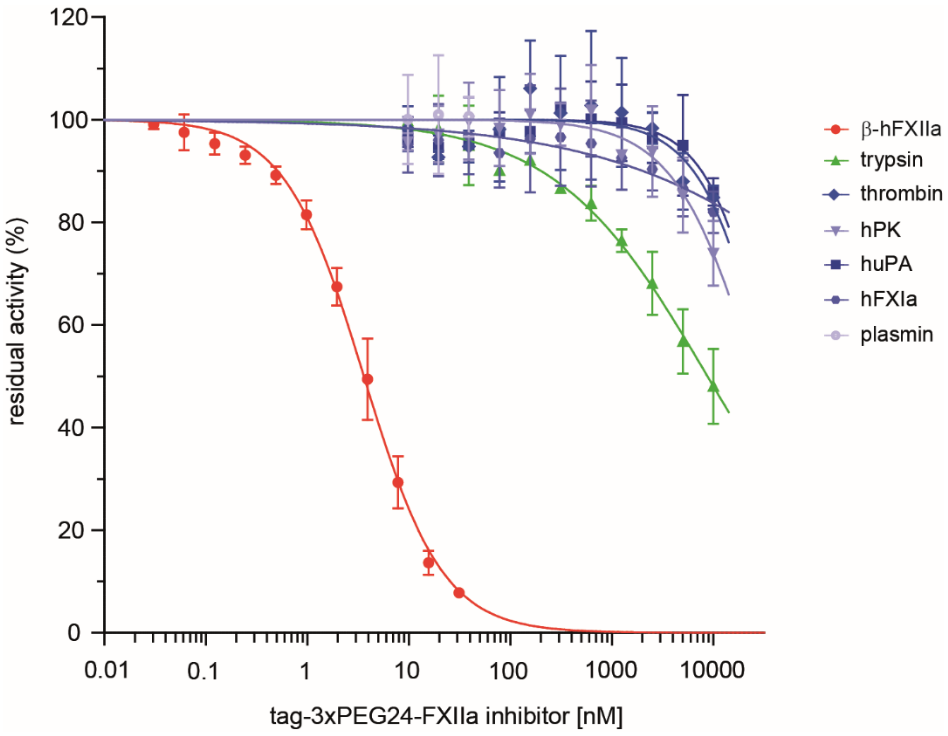

Supplementary Figure 19. Target specificity of tag-3xPEG24-FXIIa inhibitor. The residual activity of FXIIa and the indicated six homologous proteases was measured at increasing conjugate concentration using fluorogenic protease substrates. Average values and standard deviations of three measurements are shown.

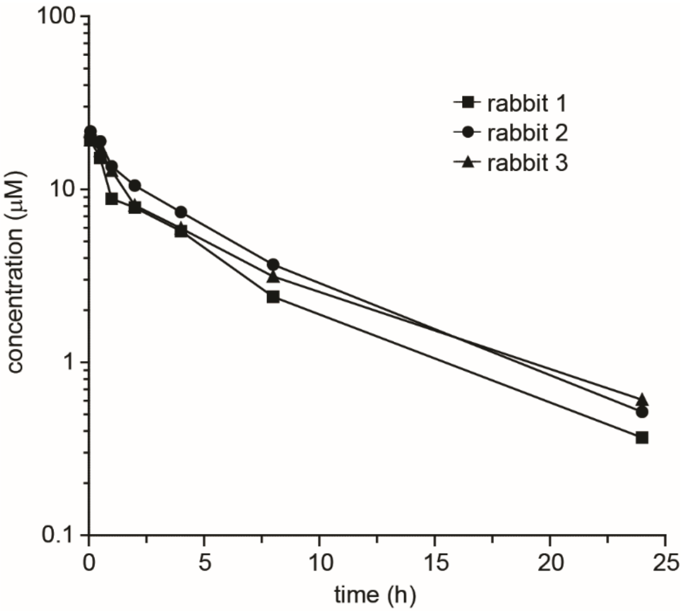

Supplementary Figure 20. Pharmacokinetic study of tag-3xPEG24-FXIIa inhibitor in rabbits. Concentration of FXIIa inhibitor in rabbit plasma after i.v. administration of 5 mg kg^{-1} tag-3xPEG24-FXIIa inhibitor.
